# Biomarkers for antimicrobial stewardship: a reappraisal in COVID-19 times?

**DOI:** 10.1186/s13054-020-03291-w

**Published:** 2020-10-06

**Authors:** Miranda van Berkel, Matthijs Kox, Tim Frenzel, Peter Pickkers, Jeroen Schouten, on behalf of the RCI-COVID-19 study group, Miranda van Berkel, Matthijs Kox, Tim Frenzel, Peter Pickkers, Jeroen Schouten, Denise Waanders, Niklas Bruse, Emma Kooistra, Hugo Touw, Pleun Hemelaar, Remi Beunders, Johannes van der Hoeven, Sjef van der Velde, Hetty van der Eng, Noortje Rovers, Margreet Klop-Riehl, Jelle Gerretsen, Nicole Waalders, Wout Claassen, Hidde Heesakkers, Tirsa van Schaik, Mihai Netea, Leo Joosten, Nico Janssen, Inge Grondman, Aline de Nooijer, Quirijn de Mast, Martin Jaeger, Ilse Kouijzer, Helga Dijkstra, Heidi Lemmers, Reinout van Crevel, Josephine van de Maat, Gerine Nijman, Simone Moorlag, Esther Taks, Priya Debisarun, Heiman Wertheim, Joost Hopman, Janette Rahamat-Langendoen, Chantal Bleeker-Rovers, Esther Fasse, Esther van Rijssen, Manon Kolkman, Bram van Cranenbroek, Ruben Smeets, Irma Joosten

**Affiliations:** 1 Department of Laboratory Medicine, Radboud University Medical Centre, 6500 HB Nijmegen, The Netherlands; 2 Department of Intensive Care Medicine, Radboud University Medical Centre, Postbus 9101, 6500 HB Nijmegen, The Netherlands; 3 Radboud Center for Infectious Diseases, Radboud University Medical Center, 6500 HB Nijmegen, The Netherlands

**Keywords:** COVID-19, Procalcitonin, Antimicrobial stewardship

On initial presentation, differentiation between early-stage coronavirus disease 2019 (COVID-19) and classical bacterial community-acquired pneumonia can be challenging. Furthermore, COVID-19 patients may develop a hyperinflammatory phase later in their disease process, which is particularly difficult to distinguish from a secondary bacterial infection. As a consequence, 72% of COVID-19 patients receive empirical antibiotic therapy during hospital stay [1]. Antibiotic overuse undoubtedly leads to an exacerbation of another—slowly progressive—pandemic: antimicrobial resistance [2].

Procalcitonin (PCT) has proven useful in the early diagnosis of lower respiratory tract infections of bacterial origin [3]. Furthermore, in the ICU setting, serial measurement of PCT can safely guide the withdrawal of antibiotic therapy [4].

In patients with COVID-19, C-reactive protein (CRP) is usually increased on presentation while PCT is often low [5]. PCT appears to increase in COVID patients with severe disease and/or in those presenting with secondary bacterial infections [6]. Longitudinal data on both biomarkers in COVID-19 infections are currently lacking. Also, it is unclear to what extent PCT and CRP predict the occurrence of secondary infections in these patients.

Data from 66 COVID-19 ICU patients were recorded in the Good Clinical Practice (GCP)-compliant data management system Castor (Castor EDC, Amsterdam, The Netherlands). PCT was determined using the Elecsys BRAHMS procalcitonin assay (Thermo Fisher Scientific), whereas CRP was determined using an immunoturbidimetric assay, both on a Cobas 8000 immunoanalyzer (Roche Diagnostics). Secondary infection was defined as “any infectious episode” evidenced by the presence of positive cultures and time-stamped at the day the culture was performed. Infectious episodes were independently determined by two ICU physicians (JS and HT). In case of incongruency, a third ICU physician (PP) was consulted. In case of multiple secondary infections, only the first infectious episode was analyzed.

Half of the patients (*n* = 33) developed a secondary infection during ICU admission. No significant differences in characteristics were observed between patients who did or did not develop a secondary infection (Table 1). In patients without secondary infection, both PCT and CRP decreased over time (Fig. 1a), with PCT values lower (peak geometric mean [95% CI] of 0.64 [0.32–1.27] μg/L) than CRP (peak geometric mean [95% CI] of 192 [107–342] mg/L) compared to their respective cutoff values for bacterial infection (< 0.5 μg/L and < 100 mg/L, respectively). A significant increase in both PCT and CRP levels was observed in case of the occurrence of a secondary infection (Fig. 1b). The receiver operating curve analysis of PCT and CRP yielded AUCs of 0.80 and 0.76, respectively (Fig. 1c). In patients with PCT < 0.25 μg/L, the negative predictive value was 81%, whereas PCT levels of > 1.00 μg/L had a positive predictive value of 93%. Intermediate PCT levels were of limited diagnostic value. For CRP, predictive values were less robust (Fig. 1c).

**Table 1:** Patient characteristics

	Secondary infection (*n* = 33)	No secondary infection (*n* = 33)	*p* value
**Sex**			
Male, *n* (%)	26 (79%)	23 (70%)	0.57
Female, *n* (%)	7 (21%)	10 (30%)	
Age, years	67 [60–73]	65 [56–70]	0.17
BMI, kg/m^2^	27.6 [25.4–31.1]	27.7 [24.3–30.7]	0.40
APACHE II	15 [13–19]	15 [10–19]	0.77
Days between COVID-19 symptoms and hospital admission	7 [4–10]	7 [5–11]	0.72
Days between COVID-19 symptoms and ICU admission	10 [7–13]	10 [6–14]	0.96
Day of secondary infection (relative to hospital admission)	16 [13–22]	NA	
Day of secondary infection (relative to ICU admission)	14 [9–21]	NA	
**Medical history,** ***n*** **(%)**			
Cardiovascular insufficiency	9 (27%)	9 (27%)	1.00
Hypertension	17 (52%)	16 (48%)	1.00
Respiratory insufficiency	2 (6%)	3 (9%)	1.00
Renal insufficiency	0 (0%)	1 (3%)	1.00
Metastatic neoplasm	2 (6%)	3 (9%)	1.00
Immunological insufficiency	0 (0%)	1 (3%)	1.00
COPD	3 (9%)	3 (9%)	1.00
Diabetes	11 (33%)	4 (12%)	0.08
Hematologic malignancy	0 (0%)	1 (3%)	1.00

Continuous data are presented as median [interquartile range]. *p* values were calculated using Mann-Whitney *U* tests (continuous data) and Fisher exact tests (categorical data)

*NA* not applicable

**Fig. 1:**
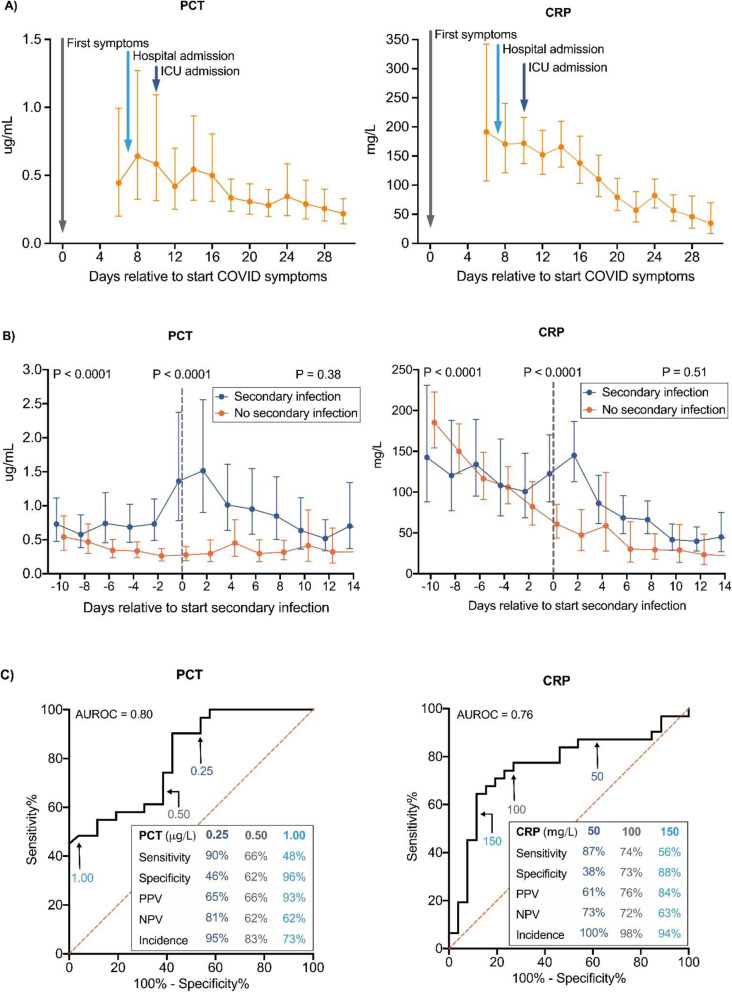
Kinetics and predictive value of procalcitonin and C-reactive protein in COVID-19 patients. **a** Serial values of procalcitonin (PCT, left panel) and C-reactive protein (CRP, right panel) in patients with COVID-19 who did not develop a secondary infection (*n* = 33). Data are aligned on the day of the start of COVID symptoms, which is designated day 0. The arrows indicate the median day of hospital admission (day 7, interquartile range [5–11]) and the median day of ICU admission (day 10, interquartile range [6–14]). Data are expressed as geometric means with 95% confidence interval. When biomarker variables were not measured daily, data were binned into bins spanning 2 days using a custom script made in R-studio v3.6.2 (www.r-project.org). If more than one value was present in these 2-day bins, the mean value was used. **b** Serial values of PCT (left panel) and CRP (right panel) in patients (*n* = 33) with COVID-19 who did develop (*n* = 33) or did not (*n* = 33) develop a secondary infection. Data are aligned on the day of secondary infection, which is designated day 0. For the “no secondary infection” group, data are aligned on the median day of infection in the “secondary infection” group (day 14 after ICU admission). Data are expressed as geometric means with 95% confidence interval. Data were binned into bins spanning 2 days (see **a** for details). The groups were compared using mixed-models analysis (time × group interaction factor) on log-transformed data. *p* values placed under graph titles reflect between-group differences over the entire time period (day − 10 until day 14). *p* values for day − 10 until day 0 and day 0 until day 14 are shown on the top left and right. **c** Receiver operating curve to illustrate sensitivity and specificity of PCT (left panel) and CRP (right panel) levels to predict secondary infection. Binned PCT/CRP data of day − 1 and day 0 were used (see **a** for details). Positive predictive value (PPV), negative predictive value (NPV), and incidence are provided for the depicted concentrations

The use of biomarkers to predict secondary infections in ICU patients warrants reappraisal in times of COVID-19. We demonstrate that COVID-19 patients who do not develop a bacterial infection present with high initial CRP levels and low-moderate PCT levels that gradually decrease over time. Furthermore, our data show that, during ICU admission, PCT levels of > 1.00 μg/L rule in, whereas concentrations of < 0.25 μg/L rule out secondary bacterial infections with good predictive values.

With regard to ICU antimicrobial stewardship, initiation of empirical antibacterial therapy in ICU patients with low PCT levels should probably not be started. As CRP is consistently elevated, this biomarker does not have predictive value for bacterial infections in the initial phase of COVID-19. Later on during ICU stay, serial PCT and, to a lesser extent, CRP may help to identify or rule out nosocomial bacterial infections and prompt appropriate use of antibiotic therapy.

## Data Availability

The datasets used and/or analyzed during the current study are available from the corresponding author on reasonable request.

## Abbreviations

COVID-19: Coronavirus disease 2019
PCT: Procalcitonin
CRP: C-reactive protein
